# Beyond Addiction: Burden of Polypharmacy and Risk in Frail Patients with Substance Use Disorder

**DOI:** 10.3390/pharmacy14010004

**Published:** 2026-01-01

**Authors:** L. Goretti Santiago Gutiérrez, Daida Alberto Armas, Verónica Hernández García, Juan Ramón Santana Ayala, Roberto García Sánchez, Soraya Paz Montelongo, Ángel J. Gutiérrez, Arturo Hardisson de la Torre, Carmen Rubio Armendáriz

**Affiliations:** 1 Addiction Treatment Unit, San Miguel Addictions, Avenida Trinidad 57, 38204 La Laguna, Spain; 2 Environmental Toxicology and Food and Pharmacological Safety Research Group, Universidad de La Laguna, 38071 La Laguna, Spain; 3 Facultad de Ciencias Biomedicas y de la Salud, Departamento de Psicología, Universidad Europea de Canarias, 38010 La Orotava, Spain

**Keywords:** drug users, substance use disorder (SUD), opioid substitution program (OSP), drug-free program (DFP), polypharmacy, drug interactions, self-medication

## Abstract

Substance use disorder (SUD) is a chronic and clinically complex condition, frequently complicated by significant organic and psychiatric comorbidities. Most patients are polymedicated and require opioid substitution programs (OSPs). This complexity is further exacerbated by drug–drug interactions, therapeutic duplication, and fragmentation of the healthcare system. This retrospective observational study analyses the prevalence of polypharmacy and associated pharmacotherapeutic risks in a cohort of 1050 patients with SUD treated at Drug Care Units (DCUs) in Tenerife (Canary Islands, Spain). Prescriptions were dominated by methadone (62%), antidepressants, and antipsychotics, often in combination with benzodiazepines. Significant polypharmacy (>10 active prescriptions) was observed in 2.3% of patients, while 8.1% received 6–10 medications and 37.2% were using 2–5 medications. Women showed a higher pharmacological burden, with 3.5% experiencing significant polypharmacy (>10 different prescriptions) compared with 1.1% of men. Overall, 31% of patients received antidepressants, 31% were treated with antipsychotics—frequently with concurrent use of multiple agents—and 6.4% received opioids outside the OSP. Therapeutic duplication was observed in 15.6% of patients for psycholeptics, 14.2% for psychoanaleptics, and 3.2% for antiepileptics. Additionally, 25.2% of patients reported self-medication, predominantly with benzodiazepines. These findings underscore the need for integrated pharmaceutical care programs incorporating individualized therapeutic review and deprescribing strategies to enhance the safety and efficacy of SUD treatment.

## 1. Introduction

Substance use disorder (SUD), as defined by the DSM-5 [1], is a chronic and recurrent disorder involving multiple factors and symptoms, characterized by the compulsive seeking and use of a psychoactive substance. It is a disorder that progresses from impulsivity to compulsivity, in which the individual experiences a transformation from seeking pleasure (positive reinforcement) to seeking relief from anxiety or stress [2]. It is considered a brain disorder because it causes functional changes in the brain circuits involved in reward, stress, and self-control. These changes can persist long after the use of the psychoactive substance has ceased [3]. The behavioral effects of these neurobiological changes manifest themselves in repeated relapses and an intense desire to use (known as “craving”) when the person is exposed to drug-related stimuli.

The neurobiology of relapses and its prevention strategies are a central area of addiction research. Contemporary models of addiction recognize “craving” as a critical determinant of both sustained substances use and the propensity to relapse after periods of abstinence. Recent advances have made it possible to characterize more precisely the experiential factors, the specific components of the neural circuits involved, and the synaptic signaling mechanisms underlying conditioned drug-seeking behavior, uncontrollable craving, and prolonged vulnerability to relapse, thus consolidating a comprehensive neurobiological framework that guides the development of therapeutic interventions aimed at modulating these responses [4,5].

Patients with SUD present a significant clinical challenge, often requiring a high level of healthcare [6]. Their clinical situation is often exacerbated by the coexistence of multiple pathologies (both organic and psychiatric comorbidity), social factors such as isolation and limited resources, and generally fragmented healthcare models. This complexity is often exacerbated by systemic factors in healthcare, such as multiple hospital admissions, frequent visits to emergency departments, the involvement of multiple prescribers, and poor treatment adherence and follow up. In addition, multiple substance use is a common feature among these patients, which increases negative outcomes, enhances addictive effects, and complicates both diagnosis and treatment [7].

Various clinical and epidemiological studies indicate that more than half of patients with substance use disorder (SUD) have concurrent psychiatric comorbidity, including mood disorders, anxiety disorders, personality disorders, and substance-induced psychosis. This comorbidity is consistently associated with poorer clinical outcomes, greater symptom severity, lower quality of life, and increased medical complications, as well as limited access to treatment services. Furthermore, in patients with SUD and significant medical comorbidity, such as HIV infection or hepatitis B and C, the presence of psychiatric disorders further increases the risk of clinical complications and hinders compliance with and effectiveness of treatment programs, underscoring the need for comprehensive approaches that simultaneously address addictive, psychiatric, and medical aspects [8,9].

This vulnerability is compounded by specific risks, including sexually transmitted and blood-borne infections, mental health disorders, gender-based violence, and social stigmatization, all of which create additional barriers to accessing and maintaining healthcare. Although these risks affect both men and women, they tend to manifest more acutely in women due to social, economic, and cultural factors [7].

Many patients with substance use disorder (SUD) are treated in opioid substitution programs (OSP), mainly with methadone and buprenorphine [10,11,12,13]. Concomitant use of methadone or buprenorphine with psychotropic, anticonvulsant, or antiretroviral drugs increases the risk of drug–drug interactions. This risk is particularly high when these agents are combined with other opioids or benzodiazepines, which may potentiate central nervous system (CNS) depression and increase the risk of overdose [11,12,14,15]. The background of the opioid crisis in the United States should not be overlooked, with the ongoing fentanyl crisis representing a particularly alarming example [16,17,18,19,20]. Furthermore, although drug interactions with newer generation antiretrovirals are less frequent and generally less clinically relevant than those associated with older treatments, careful evaluation of potential interactions remains necessary [19], especially in contexts of polypharmacy or concomitant substance use. The misuse of benzodiazepines and opioids constitutes a significant clinical problem, particularly in vulnerable populations such as people with SUD, highlighting the need for integrated strategies for pharmacotherapeutic monitoring, deprescribing, among others, to minimize risks and optimize therapeutic outcomes. This situation is exacerbated by medication-seeking behavior (also referred to as ‘prescribing under pressure’ or ‘persistent demand for medication’), self-medication and irresponsible use of medicines [14]. Among the prescription medicines that are misused, benzodiazepines and opioids stand out.

In this scenario, patients with SUD, due to their physical, psychological, social and health vulnerabilities, fit the profile of frail polymedicated patients [14]. Therefore, polypharmacy must be addressed from both a quantitative and qualitative perspective, taking into account not only the number of medications, but also their safety and clinical appropriateness [14,15].

The Spanish National Addiction Strategy (ENA 2017–2024) [21] reinforces the institutional commitment to comprehensive and coordinated care for people with substance use disorder (SUD). Within this framework, Drug Care Units (DCUs), as specialized outpatient centers, address the clinical complexity of these patients through a transdisciplinary approach, developing individualized care plans that integrate clinical comorbidities with the specific biopsychosocial characteristics of SUD. Although scientific evidence supports the effectiveness of comprehensive and multidisciplinary models in addiction care, significant structural barriers to access and continuity of care persist. Social stigma, exclusion, lack of coordination between primary care, mental health services, and DCUs, as well as the absence of unified medical records, hinder the implementation of truly patient-centered strategies. Consequently, healthcare services often operate in parallel, with limited communication and no shared clinical criteria, which negatively affects therapeutic effectiveness, contributes to the chronicity of clinical conditions [22], and increases the risk of interactions and therapeutic duplications, as demonstrated in our results.

The objective of this study was to analyze polypharmacy as conditioning factor in the clinical management of SUD. The study population comprised patients attending four Drug Care Units (DCUs) managed by the “San Miguel Adicciones” Association on the island of Tenerife (Canary Islands), recruited from a cohort established for hepatitis C virus (HCV) screening.

## 2. Materials and Methods

### 2.1. Study Design and Ethical Approval

A retrospective, observational, descriptive, cross-sectional study was conducted. The study protocol was reviewed and approved by the Ethics Committee for Research with Medicinal Products (CEIm) of the Canary Islands University Hospital Complex (CHUC), under code CHUC_2021_63.

### 2.2. Study Setting

“San Miguel Adicciones” is a non-profit organization, declared to be of public utility, with extensive experience in the treatment of addictive behaviors in the Canary Islands. Funded by the Canary Islands Government, it is a leading institution that develops comprehensive intervention programs tailored to the needs of patients. Its DCUs provide specialized care to people with addictive disorders, regardless of whether a psychoactive substance is involved.

Since 2018, the Drug Care Units (DCUs) have been collaborating with both the Canary Islands University Hospital Complex and the Nuestra Señora de La Candelaria University Hospital. This collaboration focuses on initiatives for the detection and treatment of hepatitis C virus (HCV) infection in people with substance use disorders (SUD), in line with the World Health Organization (WHO) targets for 2030. This screening was part of the Microelimination of Hepatitis C Virus Plan (MEVIC) promoted by the Spanish Association for the Study of the Liver (AEEH), the Spanish Society of Digestive Pathology (SEPD) and the Alliance for the Elimination of Viral Hepatitis in Spain (AEHVE). This collaboration included pharmacotherapeutic follow-up activities carried out by the Drug Care Units’ pharmaceutical services of “San Miguel Adicciones” (recognized under Article 62 of Law 4/2005 on Pharmaceutical Regulation in the Canary Islands), in close coordination with both Hospital’s Pharmaceutical Services.

### 2.3. Study Population and Sample Selection

**Figure 1:**
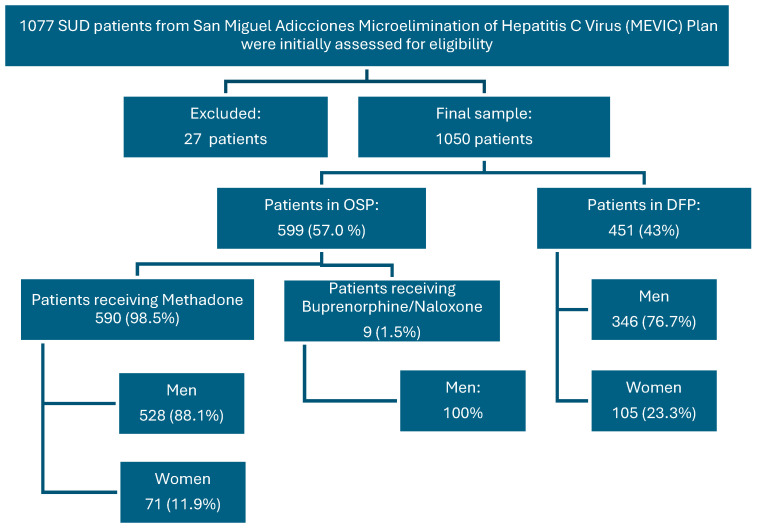
Patient selection flow chart.

All participants came from the “San Miguel Adicciones” Drug Care Units DCUs, located in the metropolitan area of the island of Tenerife, specifically in the municipalities of Santa Cruz de Tenerife and San Cristóbal de La Laguna (Figure 1).

Recruitment for this initial HCV screening was structured in successive phases, beginning with patients enrolled in the methadone Opioid Substitution Program (OSP), followed by those in the drug-free program (DFP) and, finally, including new admissions or treatment restarts.

The common inclusion criterion for HCV screening was the presence of positive HCV serology, unknown viraemia, or negative RNA for more than one year.

To be included in the present study, patients in this cohort had to meet the following criteria:

- Be 18 years of age or older.
- Have been treated at “San Miguel Adicciones” during the study period (January 2018–December 2021).
- Have participated in HCV screening as part of the MEVIC.
- Have provided signed informed consent and received the study information sheet.

Patients were excluded if their medical records contained incomplete or unreliable clinical data, as this could compromise the validity and quality of the analysis. Individuals who did not undergo the preliminary hepatitis C virus (HCV) screening, which was a prerequisite for inclusion in the study protocol, were also excluded. In addition, patients who voluntarily declined to participate or failed to provide informed consent after receiving clear, sufficient, and comprehensible information regarding the study objectives, procedures, potential benefits, and risks were not included. Such decisions were fully respected, with confidentiality ensured and without any impact on the usual healthcare provided. Finally, patients presenting significant biopsychosocial instability—defined as personal, social, or mental health factors that impeded adequate and continuous clinical follow-up throughout the study period—were excluded.

Of the 1077 patients initially considered, 27 were excluded due to incomplete or unreliable clinical information, resulting in a final study sample of 1050 patients. This cohort was distributed across two main intervention programs: 57% (*n* = 599) in the opioid substitution program (OSP) and 43% (*n* = 451) in the drug-free program (DFP). Within the OSP group, nearly all patients received methadone (*n* = 590; 98.5%), with a small proportion receiving buprenorphine/naloxone (*n* = 9; 1.5%). Gender-specific analysis revealed a higher pharmacological burden among women: while 35.3% of men (*n* = 309) were treated exclusively with methadone, this proportion was only 15.4% among women (*n* = 27) (Figure 1).

### 2.4. Data Collection and Sources

Data were collected retrospectively from multiple clinical and administrative sources to develop a complete profile of each patient. The main sources were as follows:

- Specialized electronic medical records (EMR): EMRs specific to drug addiction were reviewed to extract biopsychosocial data and information on therapeutic follow-up.
- CEDRO platform: this digital tool from the Canary Islands Government Health Service, designed for the management of addiction care, was consulted to obtain complementary sociodemographic and clinical information.
- External medical reports: further clinical data was obtained from reports issued by primary care, mental health units, hospitalization units and emergency services.
- Pharmacological history: data on medication was collected from electronic prescription systems and clinical documentation provided directly by patients.

### 2.5. Study Variables

The following variables were extracted and classified for analysis:

- Sociodemographic data: age, sex, housing and living conditions, employment status, and educational level.
- Clinical and pharmacotherapeutic data: diagnosed organic and psychiatric comorbidities, type of therapeutic program (OSP or DFP), total number of prescribed medications, use of psychotropic medications, presence of polypharmacy, reported self-medication, and concomitant use of substances with abuse potential.

### 2.6. Statistical Analysis

All data were analyzed using IBM SPSS Statistics^®^ Version 25 (IBM Corp., Armonk, NY, USA). A descriptive statistical analysis was performed. Qualitative variables were expressed as absolute and relative frequencies (*n*, %). Quantitative variables were expressed as means and standard deviations (SD).

## 3. Results

The distribution of the cohort by sex was uneven: 83% men (*n* = 875) and 17% women (*n* = 175). In terms of age, no significant differences were observed. The average age was 47 years for men and 46 years for women. Most of the SUD patients studied lived in standard housing (houses/flats) (88%), while a smaller proportion lived in institutions (municipal shelters, NGO-run shelters and transitional housing run by social organizations) (9%) or in precarious situations (uninhabitable dwellings, caves, shacks, homeless) (3%). In terms of living conditions, the most common situations were living with the family of origin (36.7%) or living alone (28.7%). The most common level of education was compulsory secondary education (43%), followed by primary education (35.2%). Only 1.8% had attained higher education. In terms of employment status, more than half of the patients were unemployed (56.8%), 22% were retired or pensioners, and only 19.7% were employed (Table 1).

**Figure 2:**
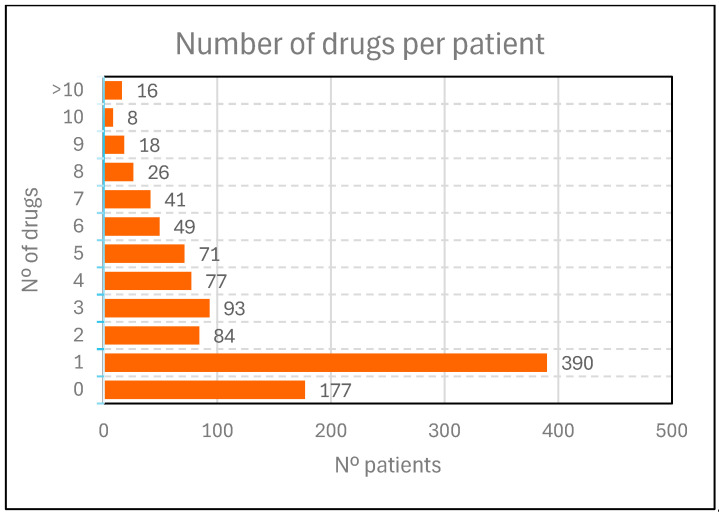
Polypharmacy among patients with SUD.

The high prevalence of polypharmacy commonly associated with SUD was confirmed in our sample (Figure 2, Table 2). Significant polypharmacy, defined as more than 10 active prescriptions, was observed in 1.8% of patients (*n* = 16). A further 16.3% (*n* = 142) received between 6 and 10 medications simultaneously, while 37.2% (*n* = 325) were prescribed 2 to 5 medications. Overall, 83.1% of patients (*n* = 873) received at least one prescribed pharmacological treatment, whereas only 16.8% (*n* = 177) were not prescribed any medication.

A more detailed analysis of the 873 patients receiving pharmacological treatment (83.1% of the total sample, Table 2) revealed distinct prescribing patterns and notable gender differences. Women were more likely than men to receive multiple prescriptions: 33.1% of women were prescribed 2–5 medications compared to 30.5% of men, while 21.1% of women received 6–10 drugs versus 12.1% of men. This disparity was even more pronounced among patients with extreme polypharmacy, with 3.5% of women prescribed more than 10 active medications compared to 1.1% of men (Table 2).

Table 3 shows the most common pharmacological treatments among patients with substance use disorder (SUD), organized according to the Anatomical Therapeutic Chemical Classification System (ATC) (36). Therapeutic duplications, defined as the simultaneous prescription of two or more drugs within the same pharmacological group, were assessed. Treatments aimed at managing addiction predominated (*n* = 648; 62%), with methadone being the most frequently prescribed active ingredient, followed by buprenorphine, bupropion, varenicline, acamprosate, and disulfiram, although in much smaller proportions. Within the psychoanaleptics group (ATC code N06), 31% of patients received antidepressants, often involving combinations of multiple active ingredients from the same subgroup, particularly selective serotonin reuptake inhibitors (SSRIs) and non-selective serotonin reuptake inhibitors. In the psycholeptics group (ATC code N05), 31% of patients were treated with antipsychotics, with frequent concurrent use of multiple agents. Significant use of antiepileptics (ATC code N03), such as valproic acid, carbamazepine, and clonazepam, was also observed; these are commonly prescribed as mood stabilizers in patients with dual disorders. Regarding analgesics, 6.4% of patients (*n* = 67) received opioids other than those used in opioid substitution programs (OSP), including morphine, oxycodone, tramadol, or fentanyl, and 22.4% of these patients received combination therapy with more than one active ingredient from the same group. The use of gabapentinoids (gabapentin and pregabalin) was also identified; these are commonly used as adjuvants for neuropathic pain but have a potential for abuse in this population.

Table 3 further illustrates the extent of therapeutic duplication within specific ATC groups. Among psychoanaleptics (ATC code N06), the highest level of duplication was observed in 10 patients (3.2%) who were prescribed three agents from the same ATC group, while 114 patients (34.7%) received two psychoanaleptics concurrently. In the psycholeptics group (ATC code N05), the greatest degree of duplication occurred in one patient (0.3%) receiving five psycholeptics, followed by six patients (1.8%) prescribed four, 38 patients (11.8%) prescribed three, and 91 patients (28.3%) prescribed two psycholeptics simultaneously. With respect to antiepileptics (ATC code N03), four patients (2.1%) received three agents from this group, and 13 patients (19.4%) received two. All proportions are expressed relative to the total number of patients treated within each pharmacological group: psycholeptics (*n* = 322), psychoanaleptics (*n* = 328), and antiepileptics (*n* = 193).

**Figure 3:**
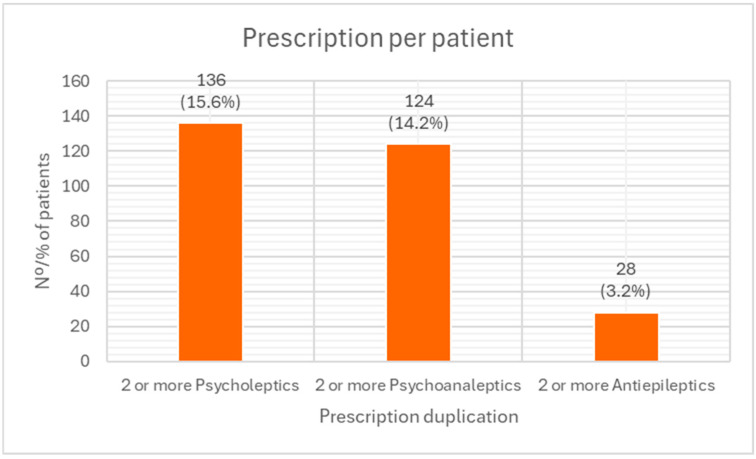
Concomitant prescribing within the same pharmacological subgroup among treated SUD patients (*n* = 873). Concomitant prescribing within the same pharmacological subgroup within the 873 SUD patients receiving pharmacological treatments.

Figure 3 shows the prevalence of the most frequent therapeutic duplications observed in patients with substance use disorder (SUD). Overall, 15.6% of patients with SUD received two or more psycholeptics (ATC code N05), 14.2% were treated with two or more psychoanaleptics (ATC code N06), and 3.2% received two or more antiepileptics (ATC code N03).

In our cohort, self-medication was reported by 25.2% of patients, predominantly involving benzodiazepines (97% of cases), particularly alprazolam, but also opioids such as tramadol and codeine.

## 4. Discussion

The study cohort showed a marked gender imbalance, with 83% male and 17% female patients, consistent with estimates from the 2023 UNODC World Drug Report [21] and the Spanish 2024 EDADES survey [18]. However, evidence indicates an upward trend in problematic substance use among women, who may develop SUD more rapidly than men [21,22]. Women not only tend to use substances less frequently, often in domestic settings or legal substances such as psychotropics and alcohol [7,23], but they are also underrepresented in treatment programs (15–25% of admissions) despite comprising a higher proportion of substance users. Barriers such as stigma contribute to this underrepresentation and are associated with greater therapeutic complexity, often due to higher psychiatric and organic comorbidity [21,24,25]. Furthermore, exposure to physical or sexual abuse significantly increases the risk of post-traumatic symptoms and SUD, particularly in women, reinforcing the need for trauma-sensitive, gender-informed interventions [26,27].

Socioeconomic vulnerability further compounds these risks. Individuals living in poverty or precarious conditions are more likely to develop and maintain substance use behaviors, creating a cycle of exclusion and persistent vulnerability that necessitates comprehensive interventions addressing social determinants of health [28,29].

Our findings indicate that over 80% of patients require pharmacological treatment, reflecting not only the management of the addictive disorder itself but also the frequent presence of comorbid psychiatric and somatic conditions. This context underscores the need for close therapeutic review to prevent drug interactions and duplicities, drug-related problems (DRPs), and potentially inappropriate prescriptions (PIPs) [30,31,32].

Therapeutic duplications were observed, particularly among psychotropic drugs. While some duplications may be justified in dual disorders or resistant symptoms, they increase the risk of interactions, adverse effects, and other medication-related events, highlighting the importance of individualized prescription review [33,34,35]. Polypharmacy within the same pharmacological subgroup, such as multiple antipsychotics or antidepressants, lacks strong evidence for additional clinical benefit and is associated with increased risk of adverse outcomes [36,37].

Self-medication constitutes a clinically relevant additional risk factor beyond the already high prevalence of prescribed polypharmacy observed in this population. When superimposed on complex pharmacotherapeutic regimens, self-medication markedly increases the likelihood of drug–drug interactions, cumulative central nervous system depression, and other drug-related problems (DRPs). In this context, self-medication should be considered a form of medication misuse, as it entails the unsupervised use of prescription-only medicines, frequently in combination with already prescribed psychotropic drugs. This additional non-prescribed drug use substantially amplifies the risk of adverse drug events and negative medication-related outcomes.

Self-medication with benzodiazepines and opioids is particularly prevalent and has been reported more frequently among women, who show higher rates of misuse [38,39]. Such misuse often involves higher-than-prescribed doses, concomitant use of multiple psychotropic agents, or concurrent alcohol consumption, thereby further increasing the risks of excessive sedation, dependence, adverse effects, and overdose. Altogether, these findings highlight the need for systematic and continuous pharmacotherapeutic review, including active screening for self-medication practices, within integrated pharmaceutical care models. In this regard, the involvement of Drug Care Units (DCUs) and specialized pharmaceutical services is essential to identify misuse, prevent DRPs, and implement individualized therapeutic optimization and deprescribing strategies aimed at improving treatment safety and clinical outcomes in patients with SUD. Moreover, these results reinforce the importance of coordinated monitoring and follow-up strategies across different levels of healthcare.

The complexity of SUD pharmacotherapy, with overlapping treatments for addiction, psychiatric comorbidities, and somatic conditions, makes structured pharmaceutical interventions essential. Drug care units and multidisciplinary teams play a crucial role in optimizing treatment safety, identifying drug related problems (DRPs), and implementing individualized review and deprescribing strategies [15,33,34,35]. Deprescribing should be approached systematically, considering scientific evidence, drug pharmacology, and patient psychosocial context, while maintaining shared decision-making and minimizing the risk of treatment discontinuation.

In conclusion, continuous, coordinated, and person-centered pharmacotherapeutic assessment is essential for SUD patients. Specialized pharmaceutical care units not only enhance treatment safety and adherence but also reduce the risks associated with polypharmacy, therapeutic duplications, and non-medical use of medications, ultimately improving clinical outcomes and the overall quality of healthcare.

## 5. Conclusions

This study highlights the high prevalence and substantial complexity of polypharmacy in patients with substance use disorder (SUD), identifying them as a highly vulnerable population with a considerable therapeutic burden. This burden is often intensified by fragmented care models, the involvement of multiple prescribers, and frequent self-medication—particularly with non-prescription benzodiazepines—creating a critical risk of drug interactions, adverse events, and other medication-related problems. Vulnerability is especially pronounced in women, who demonstrate higher rates of polypharmacy and therapeutic complexity.

The findings underscore the urgent need to integrate structured pharmaceutical care programs into the multidisciplinary management of SUD. Such programs should prioritize the identification and prevention of drug-related problems and negative drug-related outcomes, implementing individualized therapeutic review and deprescribing strategies. Continuous, person-centered pharmacotherapeutic assessment should be a fundamental component of these care models, ensuring coordinated, safe, and effective treatment.

Polypharmacy involving hypnotics, sedatives, antipsychotics, and other psychotropic drugs further amplifies the risk of drug interactions, adverse effects, and medication-related complications, reflecting the intricate pharmacological landscape faced by patients simultaneously treated for addiction, psychiatric comorbidities, and somatic conditions.

These results emphasize the critical role of specialized pharmaceutical services and drug care units in optimizing pharmacotherapy. By providing continuous monitoring, structured review, and tailored deprescribing, these services can minimize risks, enhance therapeutic efficacy, and support safer, more personalized care. Integrating such interventions within multidisciplinary teams is essential for improving clinical outcomes, reducing adverse events, and ensuring that the management of SUD is both safe and evidence-based.

## 6. Limitations

Limitations include the dynamic nature of the SUD population (affecting longitudinal representativeness), the potential underreporting bias inherent in retrospective data collection, and the incompleteness of clinical data because of fragmented health information systems.

## 7. Strengths

Strengths include the large sample size, which allows for accurate pharmacotherapeutic characterization; rigorous, contextualized data collection supported by experienced multidisciplinary teams; and the integration of the study into a nationally supported Hepatitis C Microelimination Plan, which enhances data reliability and applicability to integrated healthcare pathways.

## Figures and Tables

**Table 1:** Sociodemographic characteristics of patients with SUD.

**Gender**		**Patients (*N*)**		**Patients (%)**		**Average Age (Years)**		**Age Range (Years)**			
Males		875		83		47		20–83			
Women		175		17		46		22–75			
**Housing**											
**House/flat**				**Institution**				**Homeless/Precarious**			
*N*		%		*N*		%		*N*		%	
928		88		91		9		31		3	
**Living conditions**											
**Family of origin**		**Partner**		**Partner with children**		**Children**		**Friends**		**Single**	
*N*	%	*N*	%	*N*	%	*N*	%	*N*	%	*N*	%
385	36.7	135	12.8	139	13.3	56	5.3	33	3.2	302	28.7
**Educational level**											
**No education**		**Incomplete primary education**		**Primary education (EP)**		**Compulsory secondary education (ESO)** **(12–16 years)**		**Baccalaureate (16–18 years) or** **CFGM**		**Higher education**	
*N*	%	*N*	%	*N*	%	*N*	%	*N*	%	*N*	%
4	0.4%	38	3.6%	370	35.2	452	43.0	168	16.0	18	1.8
**Employment status**											
**Employed**			**Unemployed**			**Retired/pensioner**			**Student**		
*N*		%	N	%		*N*		%	*N*	%	
207		19.7	596	56.8		231		22.0	16	1.5	

EP: Primary Education. ESO: Compulsory Secondary Education. (12–16 years old): (equivalent to grades 7 to 10/years 8 to 11). Baccalaureate (16–18 years old): (equivalent to grades 11 and 12/A-Levels/Sixth Form), Pre-University Studies. CFGM: Vocational training courses taken after compulsory secondary education. Their aim is to provide students with technical training for the world of work, combining classroom theory with work experience in companies.

**Table 2:** Distribution of the 873 SUD patients (733 men and 140 women) receiving pharmacological treatment by number of prescribed medications, overall and by sex.

	% of the 873 Patients (Number of Patients) Using Medications	% of the 733 Men and 140 Women (Number of Patients by Sex) Using Medications	
Number of Medications per Patient		Men	Women
1 (methadone)	50.7% (336)	35.3% (309)	15.4% (27)
1 (≠methadone)	11.7% (54)	4.8% (42)	6.9% (12)
From 2 to 5	63.6% (325)	30.5% (267)	33.1% (58)
From 6 to 10	33.2% (142)	12.1% (105)	21.1% (37)
More than 10	4.6% (16)	1.1% (10)	3.5% (6)

**Table 3:** Most frequently prescribed treatments in patients with SUD.

ATC Classification (Pharmacological Subgroup)			Medication	Patients (%) and N Undergoing Treatment Out of the Total 1050	Prescription-Based Drug Duplications	
					N of Prescription-Based Drug Duplications	% (N) of the 1050 Patients Presenting Duplications
Medications indicated for addictive disorders (N02A, N06A, N07B)			Methadone	62% (648)	1	62% (648)
			Buprenorphine			
			Bupropion			
			Varenicline			
			Acamprosate			
			Disulfiram			
Psychoanaleptics (ATC code N06)	Antidepressants (ATC code N06A)	Non-selective serotonin reuptake inhibitors	Amitriptyline	31.2% (328)	1 2 3	62.1% (204) 34.7% (114) 3.2% (10)
		Selective serotonin reuptake inhibitors (SSRIs)	Paroxetine, sertraline, fluoxetine, citalopram, escitalopram, trazodone, mirtazapine, venlafaxine			
	Psychostimulants for ADHD (ATC code N06B)		Methylphenidate			
Psycholeptics (ATC code N05)	Antipsychotics (ATC code N05A)	Butyrophenone derivatives	Haloperidol	30.6% (322)	1 2 3 4 5	57.8% (186) 28.3% (91) 11.8% (38) 1.8% (6) 0.3% (1)
		Diazepine, oxazepine, thiazepine and oxepine group	Olanzapine, quetiapine, clotiapine			
		Benzamide group	Sulpiride			
		Lithium	Lithium			
		Other antipsychotics	Risperidone, aripiprazole, paliperidone			
	Anxiolytics (ATC code N05B)	Benzodiazepine derivatives	Diazepam, dipotassium clorazepate, alprazolam, lorazepam, bromazepam			
	Hypnotics and sedatives (ATC code N05C)	Benzodiazepine derivatives	Flurazepam, triazolam, lormetazepam, midazolam			
		Benzodiazepine-related	Zolpidem			
Antiepileptics (ATC code N03)		Barbiturates and derivatives	Phenobarbital	18.4% (193)	1 2 3	85.5% (165) 12.4% (24) 2.1% (4)
		Benzodiazepine derivatives	Clonazepam			
		Carboxamide derivatives	Carbamazepine, oxcarbazepine			
		Fatty acid derivatives	Valproic acid			
Analgesics (ATC code N02)		Natural alkaloids	Morphine Hydromorphone Oxycodone	6.4% (67)	1 2 3	77.6% (52) 19.4% (13) 3.0% (2)
		Phenylpiperidine derivatives	Fentanyl			
		Diphenylpropylamine derivatives	Dextropropoxyphene			
		Other opioids	Tramadol			
		Pyrazolones	Metamizole			
		Anilides	Paracetamol			
		Gabapentinoids	Gabapentin Pregabalin			

## Data Availability

The original contributions presented in this study are included in the article. Further inquiries can be directed to the corresponding authors.
